# Supplementary material for: Utilizing methylglyoxal and D-lactate in urine to evaluate saikosaponin C treatment in mice with accelerated nephrotoxic serum nephritis
Source: PLoS One. 2020 Oct 26;15(10):e0241053. doi: 10.1371/journal.pone.0241053 (PMC7588094; doi:10.1371/journal.pone.0241053)
Supplement: S1 Raw images — (PDF) [file pone.0241053.s001.pdf]

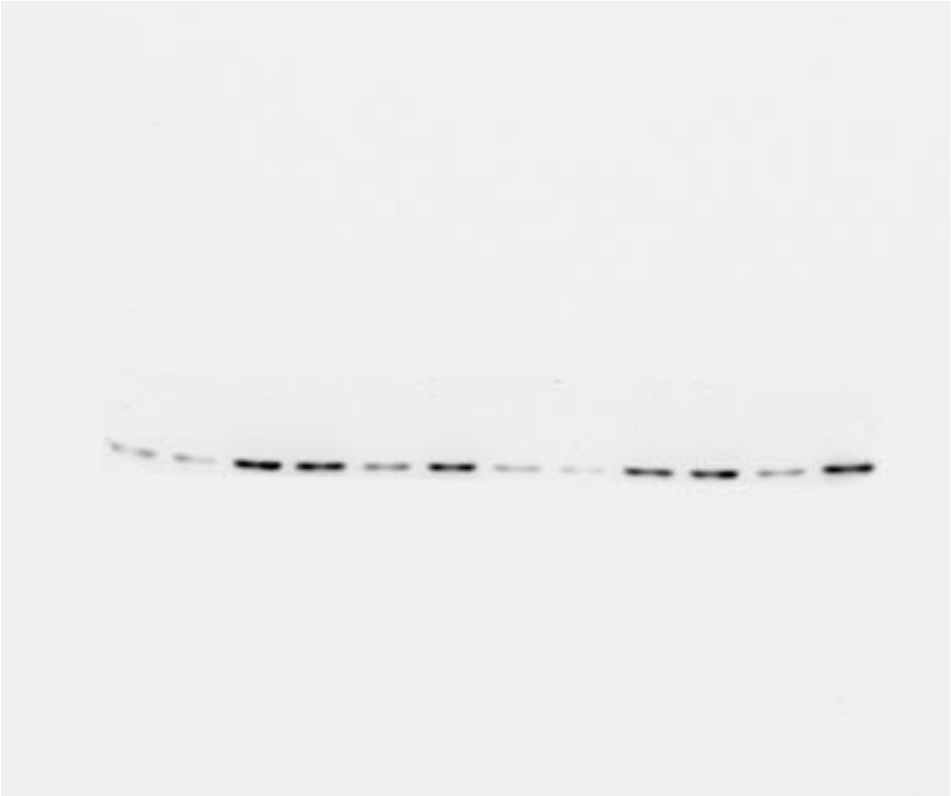

GLO 1

N1 SC1 NTS1 L1 M1 H1 N2 SC2 NTS2 L2 M2 H2

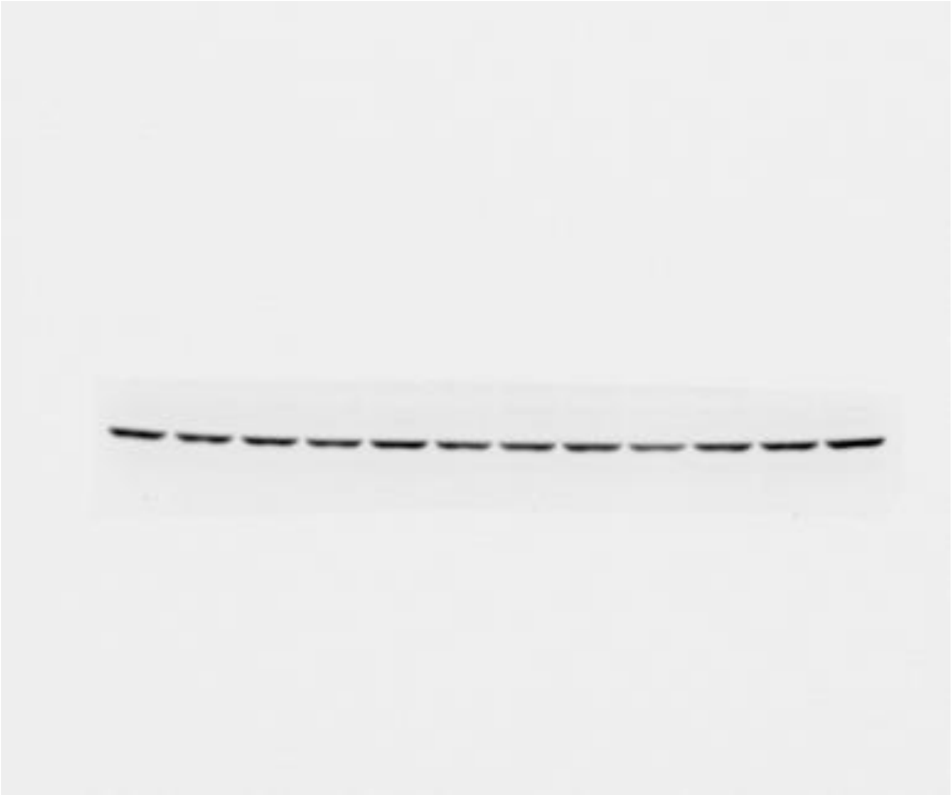

β-actin

N1 SC1 NTS1 L1 M1 H1 N2 SC2 NTS2 L2 M2 H2

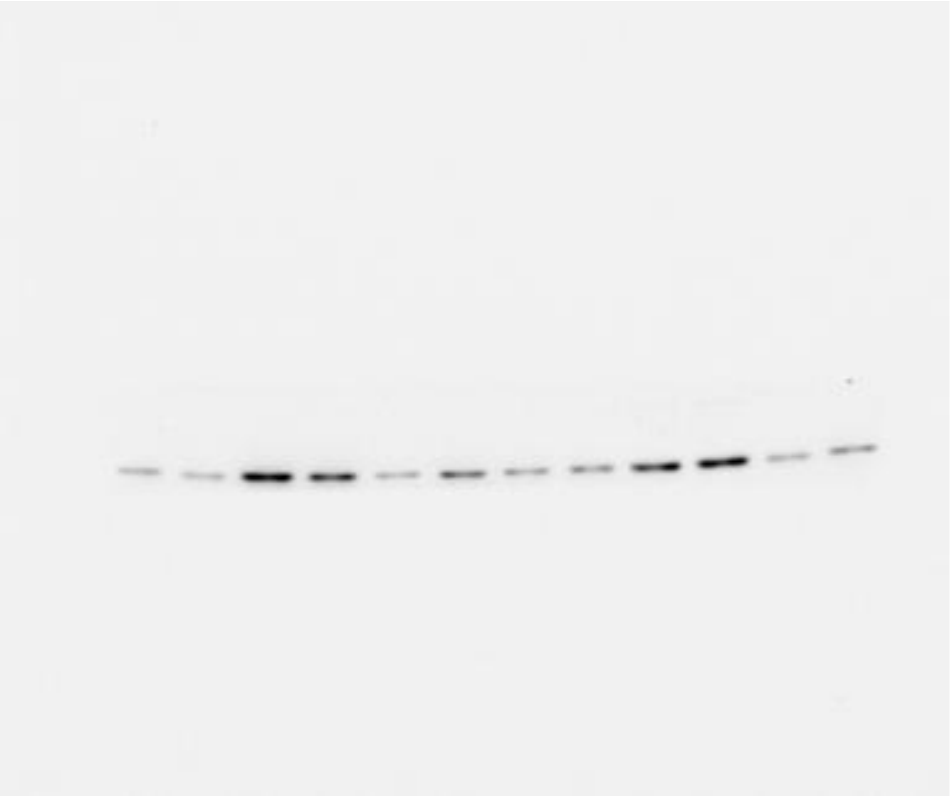

GLO 1

N3 SC3 NTS3 L3 M3 H3 N4 SC4 NTS4 L4 M4 H4

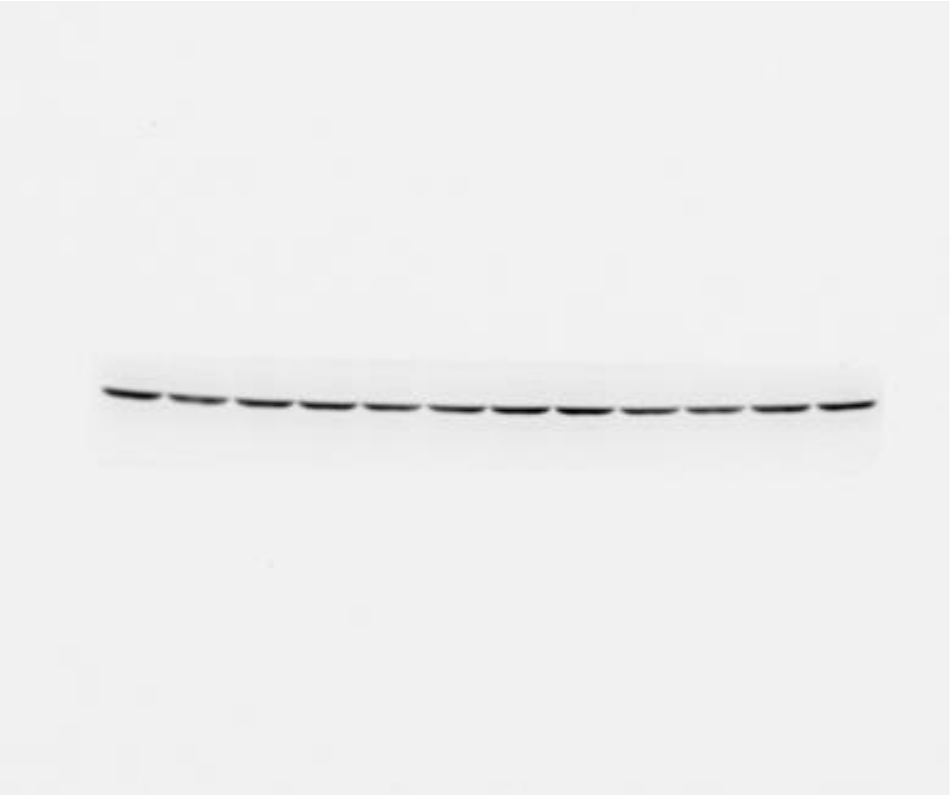

β-actin

N3 SC3 NTS3 L3 M3 H3 N4 SC4 NTS4 L4 M4 H4

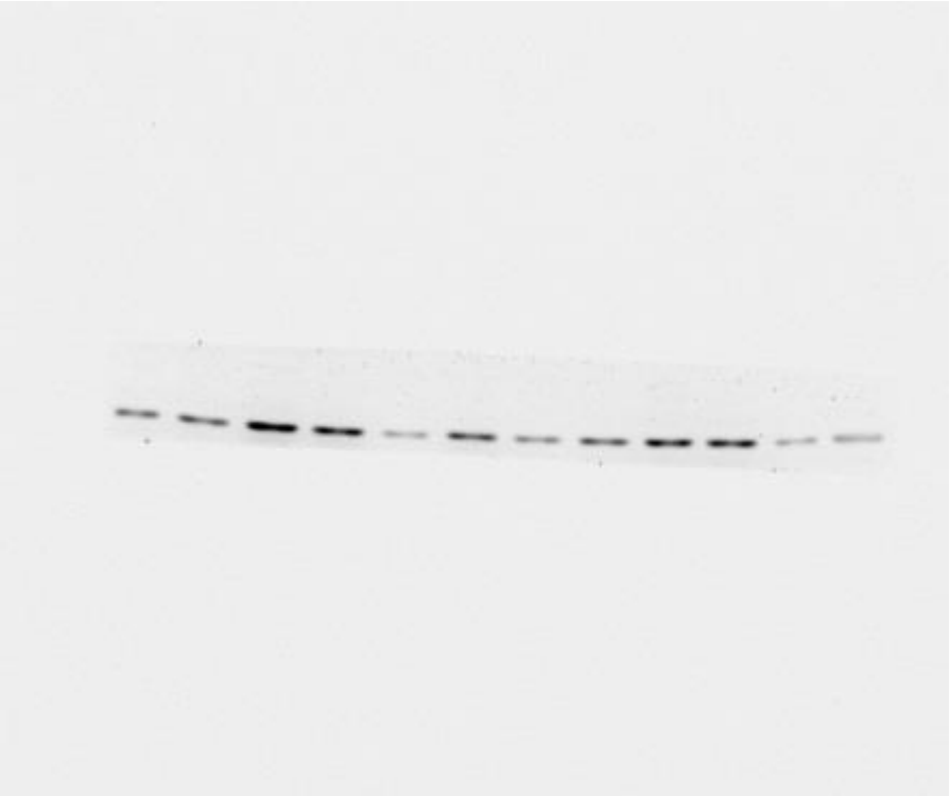

GLO 1

N5 SC5 NTS5 L5 M5 H5 N6 SC6 NTS6 L6 M6 H6

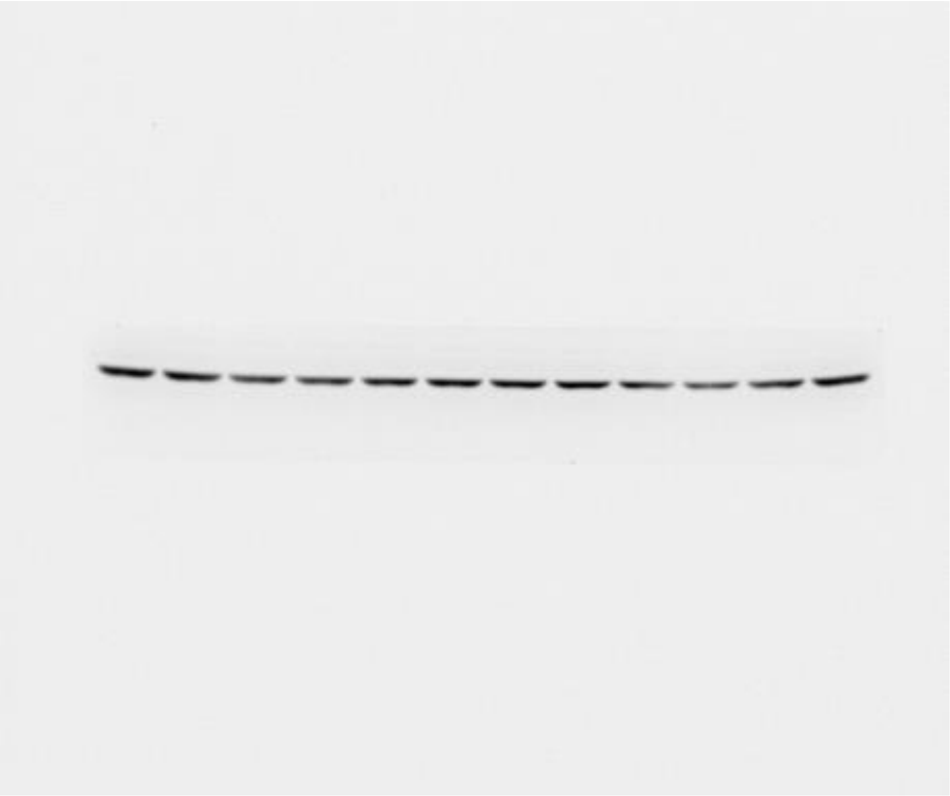

β-actin

N5 SC5 NTS5 L5 M5 H5 N6 SC6 NTS6 L6 M6 H6
