# Supplementary material for: Utilizing methylglyoxal and D-lactate in urine to evaluate saikosaponin C treatment in mice with accelerated nephrotoxic serum nephritis
Source: PLoS One. 2020 Oct 26;15(10):e0241053. doi: 10.1371/journal.pone.0241053 (PMC7588094; doi:10.1371/journal.pone.0241053)
Supplement: Attachment — Submitted filename: Response to Reviewers.docx [file pone.0241053.s002.docx]

Dear Reviewers,

Thank you for providing these insights. We have received the letter of 2020/4/28 (PONE-D-20-05825). Our research article was entitled “Utilizing methylglyoxal and D-lactate in urine to evaluate saikosaponin C treatment in mice with accelerated nephrotoxic serum nephritis by Chia-Yu Lin, Jen-Ai Lee, Po-Yeh Lin, Shih-Chun Hua, Pei-Yun Tsai, Bi-Li Chen, Chia-En Lin, Tzong-Huei Lee and Shih-Ming Chen.”. The followings are our response to your suggestion and comment. We highlighted the revised paragraph with **red color** in “Revised Manuscript with Track Changes”, so that you could figure out the the original and revised paragraph.

Reviewer #1:
Manuscript Number: PONE-D-20-05825

| Major comments | | |
| --- | --- | --- |
| Issues 1: It is somehow counterintuitive that only the intermediate, but not the high dose, of SSC is protective. This should be discussed. Potential toxic effect of the high dose should be evaluated in more detail (i.e. biochemical analysis or body weight). | | |
| Answer:  Thanks for your comments, we observed that there is no difference in SSC high dose group in serum creatinine and BUN examinations, but a high level of proteinuria in H group. It reveals that high dose of SSC may lose the protective effect from NTS nephritis due to immune-related influence. At least high dose of SSC did not perform worse than NTS injury group in present examinations. The potential toxic effect might be minor in present study.  We have added discussion as the following. | | |
| Original | | Revised |
| **Page 32, line 15 (discussion)**  ……A high dose of SSC might disrupt the balance of GR- or TNF-α- regulated systems, and counteract the anti-inflammatory benefits in nephritis. Thus, administration of high-dose SSC does not further improve the efficacy of treatment in NTS nephritis. | | **Page 38, line 7 (discussion)**  ……A high dose of SSC might disrupt the balance of GR- or TNF-α- regulated systems, and counteract the anti-inflammatory benefits in nephritis. In present study, there was no difference in high dose of SSC treatment group in serum creatinine and BUN examinations. Although the proteinuria in H group was not improved, at least it was not worse compared to NTS group. The study revealed that SSC may lose the protective effect in high dose from NTS nephritis. Thus, administration of high-dose SSC does not further improve the efficacy of treatment in NTS nephritis. |
| Issues 2: The authors state that the intermediate dose decreases the inflammatory immune infiltration and inflammation. However, these data are not shown and/or quantified. | | |
| Answer:  We have completed the ELISA analysis of TNF-α, as a quantitation of inflammation. Besides, we have added detailed items of histological score such as cells infiltration for inflammation. We revised the manuscript as the following. | | |
| **Page 13, line 19 (materials and methods)**  **ELISA analysis**  Mouse renal tumor necrosis factor α (TNF-α) level was measured using uncoated ELISA kit (Invitrogen, Carlsbad, CA, USA). All reagent preparations and experimental procedures were followed the product information sheets. | | |
| **Page 27, line 1 (results)**  **Determination of renal level of TNF-α**  Renal TNF-α measurements are shown in Fig 6. The level of TNF-α in renal tissue was significantly higher in the NTS group than the normal group, and only group M was observed significantly lower levels of TNF-α compared to the NTS group (N [0.12 ± 0.06] vs. NTS [0.30 ± 0.07]^★^ vs. M [0.16 ± 0.05]^#^; ^★^p<0.05 compared to N group, ^#^p<0.05 compared to NTS group). | | |
| **Page 28, line 1 (Fig 6)**    Fig 6. Renal TNF-α levels in the different experimental groups.  N: normal group, SC: saikosaponin C control group, NTS: nephrotoxic serum control group, L: SSC 5 mg/kg/day treatment group, M: SSC 10 mg/kg/day treatment group, H: SSC 20 mg/kg/day treatment group.  ^★^p<0.05 compared to normal group, ^#^p<0.05 compared to NTS control group. | | |
| **Page 36, line 11 (discussion)**  The trend of renal TNF-α, a biomarker of inflammation, was consistent to the histological finding. It revealed that NTS induced inflammation and damage, then caused nephritis and glomerulus sclerosis. In addition, it was observed that SSC may ameliorate the disease progression partly, although it did not work in all treatment group. | | |
| Original | | Revised |
| **Page 12, line 9 (materials and methods)**  **Histological examination**  One kidney of each mouse was embedded in paraffin and sliced into 4-5 μm sections [38]. The sections were stained with hematoxylin and eosin (H&E; Sigma-Aldrich. Inc., Saint Louis, USA), and Masson’s trichrome stain (Sigma-Aldrich. Inc., Saint Louis, USA) according to the manufacturer’s instructions. The severity of the glomerular lesions were scored on a scale of 0 (normal) to 5 (severe) in three categories (cell proliferation, thrombus, and crescent formation) as previously reported [13]. | | **Page 12, line 11 (materials and methods)**  **Histological examination**  One kidney of each mouse was embedded in paraffin and sliced into 4-5 μm sections [38]. The sections were stained with hematoxylin and eosin (H&E)~~;~~ (Sigma-Aldrich. Inc., Saint Louis, USA), ~~and~~ Masson’s trichrome stain (Sigma-Aldrich. Inc., Saint Louis, USA), and picro sirius red stain (Cis-Biotechnology Co., Ltd., Taichung, Taiwan) according to the manufacturer’s instructions. ~~The severity of the glomerular lesions were scored on a scale of 0 (normal) to 5 (severe) in three categories (cell proliferation, thrombus, and crescent formation) as previously reported [13].~~ |
| (No describing) | | **Page 13, line 2 (materials and methods)**  **Histological score**  Glomerular histological score (GHS): the severity of the glomerular lesions was scored on a scale of 0 (absent) to 5 (extensive change) in three categories (cell proliferation, thrombus, and crescent formation) as previously reported [13]. Tubulointerstitial histological score (THIS): the severity of tubulointerstitial lesions were scored on scales of 0 (absent) to 3 (extensive change) in the two categories (interstitial cells infiltration, and interstitial fibrosis), and a scale of 0 (absent) to 5 (extremely severe degeneration with massive necrosis) in the category in tubular atrophy. The total histological score (THS) was counted with the sum of GHS and THIS. |
| **Page 21, line 1 (Fig 3G)**    Fig 3. Hematoxylin and eosin stained renal tissue from different experimental groups (×200 magnification).  (A) Normal, (B) SSC control, (C) NTS control, (D) NTS + SSC 5 mg/kg, (E) NTS + SSC 10 mg/kg, and (F) NTS + SSC 20 mg/kg. (G) Histological scores for the experimental groups. ^★^p<0.05 compared to N group, ^#^p<0.05 compared to NTS control group. | | **Page 22, line 1 (Fig 3G)**    Fig 3. Hematoxylin and eosin stained renal tissue from different experimental groups (×200 magnification).  (A) Normal, (B) SSC control, (C) NTS control (arrow: the crescent), (D) NTS + SSC 5 mg/kg, (E) NTS + SSC 10 mg/kg, and (F) NTS + SSC 20 mg/kg. (G) Histological scores for the experimental groups. ^★^p<0.05 compared to N group, ^#^p<0.05 compared to NTS control group. |
| Issues 3: Mechanistic insights | | |
| Figure2: Are urine collected at the end-time point? What about proteinuria at day 15 (end of experiment)? | | |
| Answer:  We collected urine on 4 points (day-9, day-2, day5, and day12 of NTS injection), according to previous study, the severe proteinuria occurred on day 10 to 12.  (Chen SM et al., Induction of nephrotoxic serum nephritis in inbred mice and suppressive effect of colchicine on the development of this nephritis. Pharmacol Res. 2002;45(4): 319-324. https://doi.org/10.1006/phrs.2002.0948 PMID: 12030796)  (Chen SM et al., Effects of Bupleurum scorzoneraefolium, Bupleurum falcatum, and saponins on nephrotoxic serum nephritis in mice. J. Ethnopharmacol. 2008;116(3): 397-402. https://doi.org/10.1016/j.jep.2007.11.026 PMID: 18262740)  Furthermore, urine collection is a hard loading for nephritis mice, in order to reduce mortality rate, we did not collect urine on day 15 (end of experiment). | | |
| Figure 3-Figure 4  The authors describe NTS induced-damage with and histological score (defined as cell proliferation, thrombus, and crescent formation) and area of fibrosis. It would be interesting to have more information about the histological differences in mice. Are any of the analysed parameters selectively improved by the treatment with SSC? What about tubular or glomerular damage? As I have already pointed out, the authors mention differences in the inflammatory process but this is not support by any data/quantification. Staining for major immune populations should be done. | | |
| Answer:  We adding the detailed items of histological score, and revised the figure 3 and figure 4 as reviewer suggestion. And we have completed a quantitation of inflammation with TNF-α ELISA analysis as previous description. | | |
| Original | Revised | |
| **Page 12, line 9 (materials and methods)**  **Histological examination**  One kidney of each mouse was embedded in paraffin and sliced into 4-5 μm sections [38]. The sections were stained with hematoxylin and eosin (H&E; Sigma-Aldrich. Inc., Saint Louis, USA), and Masson’s trichrome stain (Sigma-Aldrich. Inc., Saint Louis, USA) according to the manufacturer’s instructions. The severity of the glomerular lesions were scored on a scale of 0 (normal) to 5 (severe) in three categories (cell proliferation, thrombus, and crescent formation) as previously reported [13]. | **Page 12, line 11 (materials and methods)**  **Histological examination**  One kidney of each mouse was embedded in paraffin and sliced into 4-5 μm sections [38]. The sections were stained with hematoxylin and eosin (H&E)~~;~~ (Sigma-Aldrich. Inc., Saint Louis, USA), ~~and~~ Masson’s trichrome stain (Sigma-Aldrich. Inc., Saint Louis, USA), and picro sirius red stain (Cis-Biotechnology Co., Ltd., Taichung, Taiwan) according to the manufacturer’s instructions. ~~The severity of the glomerular lesions were scored on a scale of 0 (normal) to 5 (severe) in three categories (cell proliferation, thrombus, and crescent formation) as previously reported [13].~~ | |
| (No describing) | **Page 13, line 2 (materials and methods)**  **Histological score**  Glomerular histological score (GHS): the severity of the glomerular lesions was scored on a scale of 0 (absent) to 5 (extensive change) in three categories (cell proliferation, thrombus, and crescent formation) as previously reported [13]. Tubulointerstitial histological score (THIS): the severity of tubulointerstitial lesions were scored on scales of 0 (absent) to 3 (extensive change) in the two categories (interstitial cells infiltration, and interstitial fibrosis), and a scale of 0 (absent) to 5 (extremely severe degeneration with massive necrosis) in the category in tubular atrophy. The total histological score (THS) was counted with the sum of GHS and THIS. | |
| (No describing) | **Page 13, line 13 (materials and methods)**  **Semi-quantitative analysis of fibrosis**  Renal fibrosis was assessed in the Masson’s trichrome and sirius red stained sections by determining the percentage area positive for aniline blue and red in ten non-overlapping fields of view for each mouse using ImageJ (National Institutes of Health, MD, USA), respectively [39]. | |
| **Page 21, line 1 (Fig 3G)**    Fig 3. Hematoxylin and eosin stained renal tissue from different experimental groups (×200 magnification).  (A) Normal, (B) SSC control, (C) NTS control, (D) NTS + SSC 5 mg/kg, (E) NTS + SSC 10 mg/kg, and (F) NTS + SSC 20 mg/kg. (G) Histological scores for the experimental groups. ^★^p<0.05 compared to N group, ^#^p<0.05 compared to NTS control group. | **Page 22, line 1 (Fig 3G)**    Fig 3. Hematoxylin and eosin stained renal tissue from different experimental groups (×200 magnification).  (A) Normal, (B) SSC control, (C) NTS control (arrow: the crescent), (D) NTS + SSC 5 mg/kg, (E) NTS + SSC 10 mg/kg, and (F) NTS + SSC 20 mg/kg. (G) Histological scores for the experimental groups. ^★^p<0.05 compared to N group, ^#^p<0.05 compared to NTS control group. | |
| **Page 22, line 1 (results)**  **Masson’s trichrome stain**  Collagen deposition surrounding the glomerulus and renal interstitium was observed in the NTS group using Masson’s trichrome staining (Fig 4C). Semi-quantitative analysis revealed a significantly percentage larger area of collagen deposition in the NTS group compared to group N, and a significantly lower percentage area of collagen deposition in group M than in the NTS group (N [1.02 ± 0.94%] vs. NTS [11.16 ± 3.02%]^★^ vs. M [4.67 ± 1.31%]#; ^★^p<0.05 compared to N group, #p<0.05 compared to NTS group; Fig 4G). Based on this experimental data, the M dosage (10 mg/kg/day) was selected as the optimal treatment dose for NTS nephritis from the three SSC groups. | **Page 23, line 1 (results)**  **Masson’s trichrome stain**  Collagen deposition surrounding the glomerulus and renal interstitium was observed in the NTS group using Masson’s trichrome staining (Fig 4C). Semi-quantitative analysis revealed a significantly percentage larger area of collagen deposition in the NTS group compared to group N, and a significantly lower percentage area of collagen deposition in group M than in the NTS group (N [1.02 ± 0.94%] vs. NTS [11.16 ± 3.02%]^★^ vs. M [4.67 ± 1.31%]#; ^★^p<0.05 compared to N group, #p<0.05 compared to NTS group; Fig 4G). ~~Based on this experimental data, the M dosage (10 mg/kg/day) was selected as the optimal treatment dose for NTS nephritis from the three SSC groups.~~ | |
| **Page 23, line 1 (results)**    Fig 4. Masson’s trichrome-stained renal tissues from different experimental groups (×200 magnification).  (A) Normal, (B) SSC control, (C) NTS control, (D) NTS + SSC 5 mg/kg, (E) NTS + SSC 10 mg/kg, and (F) NTS + SSC 20 mg/kg. (G) Quantification of the area of fibrosis for each experimental group. ^★^p<0.05 compared to normal group, #p<0.05 compared to NTS control group. | **Page 24, line 1 (results)**    Fig 4. Masson’s trichrome-stained renal tissues from different experimental groups (×200 magnification).  (A) Normal, (B) SSC control, (C) NTS control (arrow, the part of fibrosis), (D) NTS + SSC 5 mg/kg, (E) NTS + SSC 10 mg/kg, and (F) NTS + SSC 20 mg/kg. (G) Quantification of the area of fibrosis for each experimental group. ^★^p<0.05 compared to normal group, #p<0.05 compared to NTS control group. | |
| Figure5-6: Same as in figure 2. Did the authors measure these urinary parameters at end-point (day +15)?  "The appropriate dosage of saikosaponin C treatment may improve the immune-reaction and down-regulate methylglyoxal and D-lactate production"  In the discussion the authors explain the anti-inflammatory effect of SSC may be related to glucocorticoid receptor (GR) activation and inhibition of glycolysis. This is a hasty conclusion that can be true but this is not supported by any data/quantification for anti-inflammatory effect or any link with GR. | | |
| Answer:  We did not collect the urine on day 15, and the reasons are described previously.  We have quantitated TNF-alpha as inflammation barker, and anti-inflammation effect of SSC was confirmed. | | |
| Figure7:  The WB blot shown for glyoxalase 1(GLOT1) is not representative of the quantification provided. Glot1 seems more expressed in SC group compared to the N group. The GLOT1 expression looks similar in the M and H grouped. Was GLOT1 expression evaluated in all the treated mice? Additional WB image should be provided. | | |
| Answer:  We evaluated all mice, and picked one sample of each group for the WB image in figure 7. To reduce the operation error for final displayed image, we have added a new WB image with loading pooled samples of each group. The following is the new image of pooled sample. | | |
| Original | Revised | |
| **Fig 7 (GLO1 level)**   | **Fig 9 (GLO1 level)**   | |
| Minor comment | | |
| I would suggest to display individual data points in graphs (dot plot). | | |
| Answer:  Thank you for your suggestion, we have revised the figure of proteinuria, renal TNF-alpha, urinary methylglyoxal, and urinary D-lactate into dot plot as the following. | | |
| Original | Revised | |
| **Fig 2 (proteinuria)**   | **Fig 2 (proteinuria)**   | |
| (No describing) | **Fig 6 (renal TNF-alpha)**   | |
| **Fig 5 (urinary methylglyoxal)**   | **Fig 7 (urinary methylglyoxal)**   | |
| **Fig 6 (urinary D-lactate)**   | **Fig 8 (urinary D-lactate)**   | |

Reviewer #2: COMMENTS TO AUTHORS
Manuscript Number: PONE-D-20-05825

| major revisions | | |
| --- | --- | --- |
| - The images must absolutely re edited clearly. The colors are too strong and the contrast difficult to understand (masson staining has the same colors than H & E!). | | |
| Answer:  Thank you for your suggestion. We have taken new images of H&E and masson trichrome stained sections with brighter aperture, it might be clearer. | | |
| Original | Revised | |
| **Page 21, line 1 (Fig 3A to F)**    Fig 3. Hematoxylin and eosin stained renal tissue from different experimental groups (×200 magnification).  (A) Normal, (B) SSC control, (C) NTS control, (D) NTS + SSC 5 mg/kg, (E) NTS + SSC 10 mg/kg, and (F) NTS + SSC 20 mg/kg. (G) Histological scores for the experimental groups. ^★^p<0.05 compared to N group, ^#^p<0.05 compared to NTS control group. | **Page 22, line 1 (Fig 3A to F)**    Fig 3. Hematoxylin and eosin stained renal tissue from different experimental groups (×200 magnification).  (A) Normal, (B) SSC control, (C) NTS control (arrow: the crescent), (D) NTS + SSC 5 mg/kg, (E) NTS + SSC 10 mg/kg, and (F) NTS + SSC 20 mg/kg. (G) Histological scores for the experimental groups. ^★^p<0.05 compared to N group, ^#^p<0.05 compared to NTS control group. | |
| **Page 23, line 1 (Fig 4A to F)**    Fig 4. Masson’s trichrome-stained renal tissues from different experimental groups (×200 magnification).  (A) Normal, (B) SSC control, (C) NTS control, (D) NTS + SSC 5 mg/kg, (E) NTS + SSC 10 mg/kg, and (F) NTS + SSC 20 mg/kg. (G) Quantification of the area of fibrosis for each experimental group ^★^p<0.05 compared to normal group, ^#^p<0.05 compared to NTS control group. | **Page 24, line 1 (Fig 4A to F)**    Fig 4. Masson’s trichrome-stained renal tissues from different experimental groups (×200 magnification).  (A) Normal, (B) SSC control, (C) NTS control (arrow, the part of fibrosis), (D) NTS + SSC 5 mg/kg, (E) NTS + SSC 10 mg/kg, and (F) NTS + SSC 20 mg/kg. (G) Quantification of the area of fibrosis for each experimental group. ^★^p<0.05 compared to normal group, ^#^p<0.05 compared to NTS control group. | |
| - I suggest the evaluation of fibrosis using Sirius red staining. | | |
| Answer:  We have completed the evaluation of sirius red staining as reviewer suggestion, the trend of renal fibrosis is similar to masson staining. | | |
| Original | Revised | |
| **Page 12, line 9 (materials and methods)**  **Histological examination**  One kidney of each mouse was embedded in paraffin and sliced into 4-5 μm sections [38]. The sections were stained with hematoxylin and eosin (H&E; Sigma-Aldrich. Inc., Saint Louis, USA), and Masson’s trichrome stain (Sigma-Aldrich. Inc., Saint Louis, USA) according to the manufacturer’s instructions. The severity of the glomerular lesions were scored on a scale of 0 (normal) to 5 (severe) in three categories (cell proliferation, thrombus, and crescent formation) as previously reported [13]. | **Page 12, line 11 (materials and methods)**  **Histological examination**  One kidney of each mouse was embedded in paraffin and sliced into 4-5 μm sections [38]. The sections were stained with hematoxylin and eosin (H&E)~~;~~ ( Sigma-Aldrich. Inc., Saint Louis, USA), and Masson’s trichrome stain (Sigma-Aldrich. Inc., Saint Louis, USA), and picro sirius red stain (Cis-Biotechnology Co., Ltd., Taichung, Taiwan) according to the manufacturer’s instructions. ~~The severity of the glomerular lesions were scored on a scale of 0 (normal) to 5 (severe) in three categories (cell proliferation, thrombus, and crescent formation) as previously reported [13].~~ | |
| **(No describing)** | **Page 13, line 13 (materials and methods)**  **Semi-quantitative analysis of fibrosis**  Renal fibrosis was assessed in the Masson’s trichrome and sirius red stained sections by determining the percentage area positive for aniline blue and red in ten non-overlapping fields of view for each mouse using ImageJ (National Institutes of Health, MD, USA), respectively [39]. | |
| **Page 22, line 1 (results)**  **Masson’s trichrome stain**  Collagen deposition surrounding the glomerulus and renal interstitium was observed in the NTS group using Masson’s trichrome staining (Fig 4C). Semi-quantitative analysis revealed a significantly percentage larger area of collagen deposition in the NTS group compared to group N, and a significantly lower percentage area of collagen deposition in group M than in the NTS group (N [1.02 ± 0.94%] vs. NTS [11.16 ± 3.02%]^★^ vs. M [4.67 ± 1.31%]^#^; ^★^p<0.05 compared to N group, ^#^p<0.05 compared to NTS group; Fig 4G). Based on this experimental data, the M dosage (10 mg/kg/day) was selected as the optimal treatment dose for NTS nephritis from the three SSC groups. | **Page 23, line 1 (results)**  **Masson’s trichrome stain**  Collagen deposition surrounding the glomerulus and renal interstitium was observed in the NTS group using Masson’s trichrome staining (Fig 4C). Semi-quantitative analysis revealed a significantly percentage larger area of collagen deposition in the NTS group compared to group N, and a significantly lower percentage area of collagen deposition in group M than in the NTS group (N [1.02 ± 0.94%] vs. NTS [11.16 ± 3.02%]^★^ vs. M [4.67 ± 1.31%]^#^; ^★^p<0.05 compared to N group, ^#^p<0.05 compared to NTS group; Fig 4G). ~~Based on this experimental data, the M dosage (10 mg/kg/day) was selected as the optimal treatment dose for NTS nephritis from the three SSC groups.~~ | |
| **(No describing)** | **Page 25, line 1 (results)**  **Sirius red stain**  Collagen deposition was also observed in the NTS group using sirius red staining (Fig 5C). Semi-quantitative analysis revealed a significantly larger area of collagen deposition in the NTS group compared to group N, and a lower area of collagen deposition in group M than in the NTS group (N [1.38 ± 0.36%] vs. NTS [10.13 ± 4.16%]^★^ vs. M [5.25 ± 2.38%]^#^; ^★^p<0.05 compared to N group, ^#^p<0.05 compared to NTS group; Fig 5G). Based on these experimental data, the M dosage (10 mg/kg/day) was selected as the optimal treatment dose for NTS nephritis from the three SSC groups. | |
| **(No describing)** | **Page 26, line 1 (Fig 5)**    Fig 5. Sirius red-stained renal tissues from different experimental groups (×200 magnification).  (A) Normal, (B) SSC control, (C) NTS control (arrow, the part of fibrosis), (D) NTS + SSC 5 mg/kg, (E) NTS + SSC 10 mg/kg, and (F) NTS + SSC 20 mg/kg. (G) Quantification of the area of fibrosis for each experimental group. ^★^p<0.05 compared to normal group, ^#^p<0.05 compared to NTS control group. | |
| - The clear explanation of the histological score is mandatory! | | |
| Answer:  We adding the details of histological score, and revised the figure 3 as reviewer suggestion. | | |
| Original | | Revised |
| **Page 12, line 19 (Materials and Methods)**  **Histological examination**  One kidney of each mouse was embedded in paraffin and sliced into 4-5 μm sections [38]. The sections were stained with hematoxylin and eosin (H&E; Sigma-Aldrich. Inc., Saint Louis, USA), and Masson’s trichrome stain (Sigma-Aldrich. Inc., Saint Louis, USA) according to the manufacturer’s instructions. The severity of the glomerular lesions were scored on a scale of 0 (normal) to 5 (severe) in three categories (cell proliferation, thrombus, and crescent formation) as previously reported [13]. | | **Page 12, line 11 (Materials and Methods)**  **Histological examination**  One kidney of each mouse was embedded in paraffin and sliced into 4-5 μm sections [38]. The sections were stained with hematoxylin and eosin (H&E)~~;~~( Sigma-Aldrich. Inc., Saint Louis, USA), ~~and~~ Masson’s trichrome stain (Sigma-Aldrich. Inc., Saint Louis, USA), and picro sirius red stain (Cis-Biotechnology Co., Ltd., Taichung, Taiwan) according to the manufacturer’s instructions. ~~The severity of the glomerular lesions were scored on a scale of 0 (normal) to 5 (severe) in three categories (cell proliferation, thrombus, and crescent formation) as previously reported [13].~~ |
| **(No describing)** | | **Page 13, line 2 (Materials and Methods)**  **Histological score**  Glomerular histological score, GHS: the severity of the glomerular lesions were scored on a scale of 0 (absent) to 5 (extensive change) in three categories (cell proliferation, thrombus, and crescent formation) as previously reported [13]. Tubulointerstitial histological score, THIS: the severity of tubulointerstitial lesions were scored on scales of 0 (absent) to 3 (extensive change) in the two categories (interstitial cells infiltration, and interstitial fibrosis), and a scale of 0 (absent) to 5 (extremely severe degeneration with massive necrosis) in the category in tubular atrophy. The total histological score (THS) was counted with the sum of GHS and THIS. |
| Fig 3G (total histological score)   | | Fig 3G (total histological score)   |
| - As correctly reported by the authors, probably the degree of proteinuria reflects a podocyte damage. In order to evaluate it and increase the power of the result presented I suggest the evaluation of podocyte marker (f.i. nephrin, synaptopodin) in immunofluorescence of (better) in PCR. | | |
| Answer:  We are sorry for that our samples are not enough for immunofluorescence evaluation, but we provide additional reference to support that the relationship between the proteinuria severity and podocytes injury in the NTS nephritis model.  (Lin *et al.,* Decay-accelerating factor confers protection against complement-mediated podocyte injury in acute nephrotoxic nephritis. Lab Invest. 2002;82(5):563‐569. https://doi:10.1038/labinvest.3780451 PMID: 12003997)  Besides, we revised the discussion for podocytes as the following. | | |
| Original | Revised | |
| **Page 30, line 10 (results)**  Therefore, the proteinuria observed in the present study may indicated the presence of injured podocytes and glomerular dysfunction. | **Page 35, line 10 (results)**  Feng *et al.* reported that the severity of NTS proteinuria is positively related to podocyte injury. In the electronic microscopic examination, the podocytes showed extensive flattening and fusion in mice those occurred proteinuria [49]. Therefore, the proteinuria observed in the present study may indicated the presence of injured podocytes and glomerular dysfunction. | |

I appreciated very much that reviewers gave us a practical suggestion and comment to our work. Thank you very much!

Sincerely yours,

Shih-Ming Chen, Ph.D.

School of Pharmacy, College of Pharmacy,

Taipei Medical University,

No. 250, Wuxing St., Taipei 11031, Taiwan

Tel: 886-2-2736-1661 ext. 6176

Fax: 886-2-2736-6518

E-mail: smchen@tmu.edu.tw
