# Supplementary material for: Utilizing methylglyoxal and D-lactate in urine to evaluate saikosaponin C treatment in mice with accelerated nephrotoxic serum nephritis
Source: PLoS One. 2020 Oct 26;15(10):e0241053. doi: 10.1371/journal.pone.0241053 (PMC7588094; doi:10.1371/journal.pone.0241053)
Supplement: Attachment — Submitted filename: Response to Reviewers.docx [file pone.0241053.s003.docx]

Dear Reviewers,

Thank you for providing these insights. We have received the letter of 2020/8/7 (PONE-D-20-05825). Our research article was entitled “Utilizing methylglyoxal and D-lactate in urine to evaluate saikosaponin C treatment in mice with accelerated nephrotoxic serum nephritis by Chia-Yu Lin, Jen-Ai Lee, Po-Yeh Lin, Shih-Chun Hua, Pei-Yun Tsai, Bi-Li Chen, Chia-En Lin, Tzong-Huei Lee and Shih-Ming Chen.”. The followings are our response to your suggestion and comment. We highlighted the revised paragraph with **red color** in “Revised Manuscript with Track Changes”, so that you could figure out the original and revised paragraph.

Reviewer #1:
Manuscript Number: PONE-D-20-05825

| Reviewer comments | | | |
| --- | --- | --- | --- |
| The potential toxicity of the high dose is discussed, but no data shown to support their statement. An “immune-related influence” is suggested but, again, no supporting data.  The inflammatory infiltrate quantification is now provided based on a histological score. Specific immunostaining of inflammatory cells would have been preferable and more representative. | | | |
| Answer:  Thanks for your comments, we have finished the immunostaining of f4/80, marker of macrophage, and the semi-quantitation has been completed.  The trends of macrophage infiltration in each group were similar to other pathological evaluations.  We have added description in methods, results and discussion as the following. | | | |
| Original | | Revised | |
| **Page 11, line 10 (materials and methods)**    **Fig 1. Schedule of the accelerated nephrotoxic serum nephritis model.**  ……The kidneys were collected for histological examination (hematoxylin and eosin and Masson’s trichrome stain) and Immunoblotting analysis of glyoxalase 1 (GLO 1). | | **Page 11, line 11 (materials and methods)**    **Fig 1. Schedule of the accelerated nephrotoxic serum nephritis model.**  ……The kidneys were collected for histological examination (hematoxylin and eosin, ~~and~~ Masson’s trichrome, and sirius red stain), ~~and I~~immunoblotting analysis of glyoxalase 1 (GLO 1), and immunohistochemistry analysis of F4/80. | |
| (No describing) | | **Page 13, line 10 (materials and methods)**  **Immunohistochemistry analysis**  One kidney of each mouse was embedded in paraffin and sliced into 4-5 μm sections. Antibodies against F4/80 (GTX14321; GeneTex, Irvine, USA), and goat anti-rat IgG (H+L) HRP conjugate (112-035-062; Jackson ImmunoResearch Inc., PA, USA) were used at dilutions of 1:100 and 1:2000, respectively. Signals corresponding to the spots for F4/80 were measured by the Dako Liquid DAB+ substrate chromogen system (K346811; Agilent Technologies, Inc., CA, USA). | |
| **Page 13, line 9 (materials and methods)**  **Semi-quantitative analysis of fibrosis**  Renal fibrosis was assessed in the Masson’s trichrome and sirius red stained sections by determining the percentage area positive for aniline blue and red in ten non-overlapping fields of view for each mouse using ImageJ (National Institutes of Health, MD, USA), respectively [39]. | | **Page 13, line 19 (materials and methods)**  **Semi-quantitative analysis of fibrosis and macrophage infiltration**  Renal fibrosis was assessed in the Masson’s trichrome and sirius red stain, and macrophage infiltration was assessed in immunohistochemistry stain. The stained sections ~~by~~ were determined ~~determining~~ the percentage area positive for aniline blue, ~~and~~ red, and brown in ten non-overlapping fields of view for each mouse using ImageJ (National Institutes of Health, MD, USA), respectively [39]. | |
| (No describing) | | **Page 28, line 1 (results)**  **Immunohistochemistry stain**  The infiltration of macrophage was observed in the NTS group using immunohistochemistry staining (Fig 6C). Semi-quantitative analysis revealed a significantly larger area of macrophage infiltration in the NTS group compared to group N, and a less area of macrophage infiltration in group M than in the NTS group (N [ 0.068 ± 0.036 %] vs. NTS [ 1.534 ± 0.523 %]^★^ vs. M [ 0.624 ± 0.702 %]^#^; ^★^*p*<0.05 compared to N group, ^#^*p*<0.05 compared to NTS group; Fig 6G). | |
| (No describing) | | **Page 29, line 2 (results)**    **Fig 6. Immunohistochemistry stained renal tissues from different experimental groups (×200 magnification).**  (A) Normal, (B) SSC control, (C) NTS control (arrow, the part of macrophage infiltration), (D) NTS + SSC 5 mg/kg, (E) NTS + SSC 10 mg/kg, and (F) NTS + SSC 20 mg/kg. (G) Quantification of the area of fibrosis for each experimental group. ^★^*p*<0.05 compared to normal group, ^#^*p*<0.05 compared to NTS control group. | |
| **Page 36, line 11 (discussion)**  The trend of renal TNF-α, a biomarker of inflammation, was consistent to the histological finding. It revealed that NTS induced inflammation and damage, then caused nephritis and glomerulus sclerosis. In addition, it was observed that SSC may ameliorate the disease progression partly, although it did not work in all treatment group. | | **Page 39, line 12 (discussion)**  The trend of renal TNF-α, a biomarker of inflammation, was consistent to the histological finding. The NTS induced renal damage in histological observation is related to inflammation. The similar trend was observed in renal level of F4/80, a biomarker of macrophage. It revealed that ~~NTS induced inflammation and damage, then caused nephritis and glomerulus sclerosis.~~ the level of macrophage infiltration induced by NTS injection is positively correlated with nephritis severity as previous reports noted [50, 51]. Besides, the depletion of macrophage may improve disease in several animal models of autoimmune related nephritis [50, 51, 52]. In addition, it was observed that SSC may partly ameliorate the disease progression ~~partly~~ and decrease the level of macrophage infiltration, although it did not work in all treatment ~~group~~ groups. | |
| Issues 2:  Figure 3-4 and 5 please ensure that the magnification is the same for all panels. Scale bars are not provided for figure. | | | |
| Answer:  The magnification for all panels is the same, and we have added scale bars for all pathological pictures as the following. | | | |
| Original | | | Revised |
| **Page 22, line 1 (Fig 3)**    **Fig 3. Hematoxylin and eosin stained renal tissue from different experimental groups (×200 magnification).** | | | **Page 23, line 1 (Fig 3)**    **Fig 3. Hematoxylin and eosin stained renal tissue from different experimental groups (×200 magnification).** |
| **Page 24, line 1 (Fig 4)**    **Fig 4. Masson’s trichrome-stained renal tissues from different experimental groups** | | | **Page 25, line 1 (Fig 4)**    **Fig 4. Masson’s trichrome-stained renal tissues from different experimental groups** |
| **Page 26, line 1 (Fig 5)**    **Fig 5. Sirius red-stained renal tissues from different experimental groups (×200 magnification).** | | | **Page 27, line 1 (Fig 5)**    **Fig 5. Sirius red-stained renal tissues from different experimental groups (×200 magnification).** |
| (No describing) | | | **Page 29, line 1 (Fig 6)**    **Fig 6. Immunohistochemistry stained renal tissues for F4/80 from different experimental groups (×200 magnification).** |
| Issues 3:  WB in Figure 7. I would have preferred to see the individual mice, not to pool them. | | | |
| We have retested the individual mice of each group again. The image of one set samples have been picked from six sets as the following. | | | |
| Original | Revised | | |
| **Page 33, line 1 (result)**  **Immunoblotting analysis**  GLO1 expression in renal tissue was significantly higher in the NTS group than the normal group (5.33 ± 0.81, p<0.05 compared to N group), and only group M expressed significantly lower levels of GLO1 compared to the NTS group (2.16 ± 0.84, p<0.05 compared to NTS group; Fig 9B). | **Page 36, line 3 (result)**  **Immunoblotting analysis**  GLO1 expression in renal tissue was significantly higher in the NTS group than the normal group (~~5.33 ± 0.81~~ 4.90 ± 2.12, *p*<0.05 compared to N group), and only group M expressed significantly lower levels of GLO1 compared to the NTS group (~~2.16 ± 0.84~~ 1.50 ± 0.47, *p*<0.05 compared to NTS group; Fig ~~9B~~ 10B). | | |
| **Page 34, line 1 (Fig 9)**    **Fig 9. Immunoblotting analysis of glyoxalase 1 in renal homogenates from different experimental groups on day 15.** | **Page 37, line 3 (Fig 10)**    **Fig ~~9~~10. Immunoblotting analysis of glyoxalase 1 in renal homogenates from different experimental groups on day 15.** | | |
| **Supplement**  **GLO1**    **N, SC, NTS, L, M, H, N, SC, NTS, M**  **β-actin**    **N, SC, NTS, L, M, H, N, SC, NTS, M** | **Supplement** **GLO1**  **N1** \| **SC1** \| **NTS1** \| **L1** \| **M1** \| **H1** \| **N2** \| **SC2** \| **NTS2** \| **L2** \| **M2** \| **H2** \| \| \| \| \| \| **β-actin**  **N1** \| **SC1** \| **NTS1** \| **L1** \| **M1** \| **H1** \| **N2** \| **SC2** \| **NTS2** \| **L2** \| **M2** \| **H2** \| \| \| \| \| \| **GLO1**  **N3** \| **SC3** \| **NTS3** \| **L3** \| **M3** \| **H3** \| **N4** \| **SC4** \| **NTS4** \| **L4** \| **M4** \| **H4** \| \| \| \| \| \| **β-actin**  **N3** \| **SC3** \| **NTS3** \| **L3** \| **M3** \| **H3** \| **N4** \| **SC4** \| **NTS4** \| **L4** \| **M4** \| **H4** \| \| \| \| \| \| **GLO1**  **N5** \| **SC5** \| **NTS5** \| **L5** \| **M5** \| **H5** \| **N6** \| **SC6** \| **NTS6** \| **L6** \| **M6** \| **H6** \| \| \| \| \| \| **β-actin**  **N5** \| **SC5** \| **NTS5** \| **L5** \| **M5** \| **H5** \| **N6** \| **SC6** \| **NTS6** \| **L6** \| **M6** \| **H6** \| \| \| \| \| \| | | |

I appreciated very much that reviewers gave us a practical suggestion and comment to our work. Thank you very much!

Sincerely yours,

Shih-Ming Chen, Ph.D.

School of Pharmacy, College of Pharmacy,

Taipei Medical University,

No. 250, Wuxing St., Taipei 11031, Taiwan

Tel: 886-2-2736-1661 ext. 6176

Fax: 886-2-2736-6518

E-mail: smchen@tmu.edu.tw
